# Identification of genomic regions associated with feed efficiency in Nelore cattle

**DOI:** 10.1186/s12863-014-0100-0

**Published:** 2014-09-26

**Authors:** Priscila SN de Oliveira, Aline SM Cesar, Michele L do Nascimento, Amália S Chaves, Polyana C Tizioto, Rymer R Tullio, Dante PD Lanna, Antonio N Rosa, Tad S Sonstegard, Gerson B Mourao, James M Reecy, Dorian J Garrick, Maurício A Mudadu, Luiz L Coutinho, Luciana CA Regitano

**Affiliations:** Department of Genetics and Evolution, Federal University of Sao Carlos, 13565-905 Sao Carlos, SP Brazil; Department of Animal Science, University of Sao Paulo, 13418-900 Piracicaba, SP Brazil; Embrapa Southeast-Cattle Research Center, 13560-970 Sao Carlos, SP Brazil; Embrapa Beef Cattle, 79002-970 Campo Grande, MS Brazil; USDA ARS Bovine Functional Genomics Laboratory, Beltsville, MD 20705 USA; Department of Animal Science, Iowa State University, Ames, IA 50011 USA

**Keywords:** *Bos indicus*, Candidate gene, Residual feed intake, Single nucleotide polymorphisms

## Abstract

**Background:**

Feed efficiency is jointly determined by productivity and feed requirements, both of which are economically relevant traits in beef cattle production systems. The objective of this study was to identify genes/QTLs associated with components of feed efficiency in Nelore cattle using Illumina BovineHD BeadChip (770 k SNP) genotypes from 593 Nelore steers. The traits analyzed included: average daily gain (ADG), dry matter intake (DMI), feed-conversion ratio (FCR), feed efficiency (FE), residual feed intake (RFI), maintenance efficiency (ME), efficiency of gain (EG), partial efficiency of growth (PEG) and relative growth rate (RGR). The Bayes B analysis was completed with Gensel software parameterized to fit fewer markers than animals. Genomic windows containing all the SNP loci in each 1 Mb that accounted for more than 1.0% of genetic variance were considered as QTL region. Candidate genes within windows that explained more than 1% of genetic variance were selected by putative function based on DAVID and Gene Ontology.

**Results:**

Thirty-six QTL (1-Mb SNP window) were identified on chromosomes 1, 2, 3, 5, 6, 7, 8, 9, 10, 12, 14, 15, 16, 18, 19, 20, 21, 22, 24, 25 and 26 (UMD 3.1). The amount of genetic variance explained by individual QTL windows for feed efficiency traits ranged from 0.5% to 9.07%. Some of these QTL minimally overlapped with previously reported feed efficiency QTL for *Bos taurus*. The QTL regions described in this study harbor genes with biological functions related to metabolic processes, lipid and protein metabolism, generation of energy and growth. Among the positional candidate genes selected for feed efficiency are: *HRH4, ALDH7A1, APOA2, LIN7C, CXADR, ADAM12* and *MAP7.*

**Conclusions:**

Some genomic regions and some positional candidate genes reported in this study have not been previously reported for feed efficiency traits in *Bos indicus*. Comparison with published results indicates that different QTLs and genes may be involved in the control of feed efficiency traits in this Nelore cattle population, as compared to *Bos taurus* cattle.

**Electronic supplementary material:**

The online version of this article (doi:10.1186/s12863-014-0100-0) contains supplementary material, which is available to authorized users.

## Background

Feed efficiency has a major influence on the unit cost of beef production. Selection of efficient animals not only improves the producer’s profitability, but can lead to significant reductions in the required pasture area per unit of production, decrease feed cost in feedlots, as well as, reduced environmental impact, through lower carbon and methane emissions [1].

Feed efficiency is typically defined as feed conversion ratio (DMI; dry matter intake divided by ADG; average daily gain), nonetheless other indexes have been studied, such as residual feed intake (RFI; [2]), maintenance efficiency (ME; [3]), partial efficiency of growth (PEG; [4]), efficiency of gain (EG) and relative growth rate (RGR; [5]). Despite its moderate heritability from 0.20 to 0.30 [6-9], feed efficiency has not been considered in Brazilian breeding programs, probably due to the difficulties associated with collection of individual feed intake data [6].

Brazil is one of the world’s leading beef producers and exporters, and the *Bos indicus* Nelore is the predominant cattle breed in Brazil. *Bos indicus* animals are known for their adaptability and resilience in tropical environments. Nevertheless, there are only a few [10] genomic studies reported for feed efficiency traits in this breed.

Genome wide association studies (GWAS) involve whole genome search for chromosomal regions that are significantly associated with phenotype, and can be used for QTL detection, determination of gene networks or genomic selection [11,12]. The detection of genomic regions and candidate genes which influence feed efficiency traits in Nelore should contribute to breeding programs. The objective of this study was to identify genes/QTLs associated with feed efficiency traits in Nelore by genome wide association study using the BovineHD BeadChip (770 k) and Bayesian statistical approaches.

## Results and discussion

### Phenotypic data

The mean values for all traits evaluated in this study (Table 1) are in agreement with the literature [13-17].

**Table 1:** **Descriptive statistics for feed efficiency traits in Nelore cattle**

**Phenotype**	**N**	**Mean ± SE**
ADG [Average Daily Gain; kg/d]	593	1.274 ± 0.34
DMI [Dry Matter Intake; kg/d]	591	8.451 ± 1.28
FCR [Feed Conversion Ratio; kg/kg]	516	7.147 ± 3.07
FE [Feed Efficiency, kg/kg]	516	0.155 ± 0.04
RFI [Residual Feed Intake, kg/d]	591	0.001 ± 0.62
ME [Maintenance Efficiency; kg/kg]	376	0.349 ± 0.13
EG [Efficiency of Gain, kg/Mcal]	376	0.207 ± 0.10
PEG [Partial Efficiency of Growth]	375	0.275 ± 0.05
RGR [Relative Growth Rate; %/d]	593	0.165 ± 0.04

### Genome wide association study

Table 2 shows mean genetic and residual variances and genomic heritability, the proportion of phenotypic variance explained by markers using Bayes B, which assumes that SNP effects have a locus-specific variance and some fraction of markers (π) have no effect [18,19].

**Table 2:** **Posterior means of variance components for feed efficiency traits in Nelore cattle**

**Phenotype**	**Genetic variance**	**Residual variance**	**Total variance**	**Proportion of variance explained by SNP**
ADG	0.06	0.08	0.15	0.42
DMI	0.52	1.22	1.75	0.29
FCR	2.96	4.53	7.50	0.39
FE	0.13	0.009	0.23	0.47
RFI	0.14	0.27	0.41	0.33
ME	0.01	0.01	0.02	0.51
EG	0.0042	0.0034	0.0076	0.55
PEG	0.0027	0.0020	0.0048	0.57
RGR	0.0024	0.0005	0.00030	0.18

ADG: average daily gain [kg/d], DMI: dry matter intake [kg/d], FCR: feed conversion ratio [kg/kg], FE: feed efficiency [kg/kg], RFI: residual feed intake [kg/d], ME: maintenance efficiency [kg/kg], EG: efficiency of gain [kg/Mcal], PEG: partial efficiency of growth, RGR: relative growth rate [%/d].

The estimated proportion of phenotypic variance explained by genome markers or “genomic heritability” for all measures of feed efficiency were moderate to high, except for RGR (Table 2). Estimated moderate heritability for feed efficiency traits are in agreement with published estimates from traditional and genomic analyses [6,8,9,20,21].

The amount of genetic variance explained by individual QTL windows for feed efficiency traits ranged from 0.5% to 9.07% (Table 3, Additional file 1: Table S1). These findings are in agreement to reported results for *Bos taurus* cattle where SNPs explained large amounts of variation in feed efficiency-related traits [22]. The largest QTLs were located on BTA2, 9 and 12 (Table 3).

**Table 3:** **QTL regions associated with feed efficiency traits in Nelore cattle**

**Phenotype**	**SNP window [start and end SNP]**	**Number SNP in window**	**% Variance explained by SNP window**	**Chr**	**Map position [UMD 3.1 bovine assembly]**	**Functional candidate genes**
ADG	rs133645581-rs137479730	231	4.94	9	22019753… 22993318	*-*
	rs134217291- rs43586239	245	2.91	1	156005880…156999077	-
	rs134861070*-*rs133661192	176	2.33	16	31007509… 31998663	-
	rs109785180*-* rs42314597	181	1.62	7	28006905… 28999205	*MARCH3, ALDH7A1*
	rs110427506-rs109577271	215	1.31	3	8007708… 8998553	*APOA2,, USP21, UFC1*
	rs135933205-rs136131764	270	1.01	14	6007610… 6998560	-
DMI	rs133031353-rs42739324	237	3.76	24	32007830…32998471	*HRH4, ZNF521*
	rs134105133-rs133615999	161	2.00	13	8009711…8998026	-
	rs133460769-rs109902312	255	1.29	9	8000493…8996276	-
FCR	rs110424374-rs133308150	73	6.06	12	27042151…27967155	-
	rs41942246-rs134122046	191	5.99	20	36002437…36994188	*GDNF*
	rs42594525-rs109404921	182	1.52	15	58000208…58999275	*LIN7C*
	rs109105703 *-*rs136356118	189	1.40	18	15002281…15989210	*-*
FE	rs133645581-rs137479730	231	2.58	9	22019753…22993318	*-*
	rs134914044-rs42277860	203	2.30	1	21002744…21985704	-
	rs136028559-rs110570158	250	1.04	14	73002666…73999191	-
	rs109171156-rs42987702	255	1.03	2	58005318…58996319	-
RFI	rs132846819-rs136767848	217	1.50	8	43001055…43994362	
	rs109535395-rs134508640	50	1.42	18	14008775… 14999189	*DEPP1, TUBB3*
	rs109365529-rs132654030	186	1.12	11	93000777… 93986308	*PTGS1*
	rs136295413-rs41980878	260	1.12	21	35004352… 35999673	-
ME	rs110886051*-*rs136158385	132	2.23	9	85000924…85992426	-
	rs135793109-rs134017120	244	1.48	14	83000515…83999590	*-*
	rs132642498-rs42290512	195	1.28	20	42002534…42999156	-
	rs136695261-rs134921622	265	1.24	1	155006277…155998842	*DPH3*
	rs41681891-rs111007160	315	1.22	13	19000011…19998017	-
	rs134217291-rs43586239	245	1.11	1	156005880…156999077	-
EG	rs135733781-rs136250718	170	3.42	10	82000451…82991463	-
	rs41681891-rs111007160	315	2.01	13	19000011…19998017	-
	rs135553767-rs133216097	265	1.73	20	58002823…58996938	-
	rs132642498-rs42290512	195	1.39	20	42002534…42999156	-
	rs134907990-rs135714341	143	1.14	28	29001640…29996795	*CAMK2G*
PEG	rs135793109- rs134017120	244	2.25	14	83000515…83999590	*-*
	rs136639955- rs109263205	208	1.63	10	27001939…27998826	-
	rs41681891 *-*rs111007160	315	1.30	13	19000011…19998017	-
	rs42442541-rs42102604	324	1.29	26	46000238…46999583	*ADAM12*
	rs137164093 *-*rs137788588	158	1.10	3	55053110…55996246	-
	rs43109937-rs135732317	102	1.08	1	18002981…18998146	*CXADR*
RGR	rs110498934*…*rs109401708	89	9.07	2	63002463… 63957289	-
	rs135507221…rs136312022	170	1.72	12	64002872… 64983441	-
	rs41576182… rs137330342	227	1.23	12	63000336… 63998631	-
	rs137740719 *…*rs132674185	236	1	9	75004071… 75998811	*MAP7, PEX7*

ADG: average daily gain [kg/d], DMI: dry matter intake [kg/d], FCR: feed conversion ratio [kg/kg], FE: feed efficiency [kg/kg], RFI: residual feed intake [kg/d], ME: maintenance efficiency [kg/kg], EG: efficiency of gain [kg/Mcal], PEG: partial efficiency of growth, RGR: relative growth rate [%/d].

Pooling results across all traits, 36 genomic regions (1-Mb windows) that explain ≥ 1% of genetic variance (Table 3) were identified in this study. These were on chromosomes: 1, 2, 3, 5, 6, 7, 8, 9, 10, 12, 14, 15, 16, 18, 19, 20, 21, 22, 24, 25 and 26. Some of these QTL minimally overlapped with previously reported feed efficiency QTL for other breeds according to QTL database (http://www.animalgenome.org/cgi-bin/QTLdb/BT/index). This outcome may indicate that different QTL may control feed efficiency traits in this Nelore population as compared to *Bos Taurus* [22-25]. Genomic regions that explained > 0.5% of genetic variance for all traits are showed in Additional file; and Additional files 2, 3, 4, 5, 6, 7, 8, 9 and 10 show Manhattan plots of proportion of genetic variance explained across the 29 autosome chromosomes for DMI, ADG, FC, FE, RFI, ME, PEG, GE and RGR, respectively.

Three genomic regions explained >1% of genetic variance for DMI (Table 3). The region on BTA24 at 32 Mb was also associated with RFI (Additional file 1: Table S1). Two positional and functional candidate genes were selected in this region. Histamine receptor H4 (*HRH4*) is the first candidate, which is associated with regulation of appetite and immune and inflammatory responses [26]. Histamine is produced by the decarboxylation of histidine by histidine decarboxylase, and it exerts its action by binding to specific histamine receptors (HRs) [27]. Histamine mediates a variety of physiological processes, which includes inflammation and immunity, gastric acid secretion, smooth muscle contraction, tissue growth and repair, food allergies and regulation of appetite and metabolism in humans [28]. The second candidate gene is zinc finger protein 521 (*ZNF521*) also found as “zinc finger” protein (*ZNF804B),* characterized by coordination and stabilization of zinc ions in several ionic exchange processes associated to DMI in Nelore cattle on BTA4 [10].

Six genomic regions explained >1% of genetic variance for ADG (Table 3). The region on BTA9 at 22 Mb was also associated with FE. On BTA7 at 28 Mb two genes with biological function related to weight gain have been annotated; membrane-associated ring finger (*MARCH3)* and aldehyde dehydrogenase 7 family, member A1 *(ALDH7A1*)*. MARCH3* gene is related to proteolysis according to gene ontology terms (GO: 0006508). Proteolysis is the hydrolysis of proteins into smaller polypeptides and/or amino acids by cleavage of their peptide bonds (Kegg). A study have indicated that rates of proteolysis and protein synthesis are greater in obese than in lean subjects, whereas others studies have not supported this findings [29]. *ALDH7A1* gene is part of a pathway related to glycolysis/gluconeogenesis (Kegg). Glycolysis is the sequence of reactions that metabolizes one molecule of glucose to two molecules of pyruvate with the concomitant production of ATP [30], and the glycolytic/gluconeogenic pathways may play an integral role in body weight regulation [31]. *ALDH7A1* gene encodes the antiquin protein that has been shown to be associated with increased body weight gain and obesity in rats [32].

On BTA3 at 8 Mb three candidate genes were identified; apolipoprotein A-II (*APOA2*)*,* ubiquitin specific peptidase 21 (*USP21*), and ubiquitin-fold modifier conjugating enzyme 1 (*UFC1*). *APOA2* gene has been assigned biological terms of protein transport (GO: 0051223), lipid metabolism (GO: 0008610) and response to carbohydrate stimulus (GO: 0009743). *APOA2* gene produces apolipoprotein A-II that is a key regulatory factor of HDL metabolism [33]. Polymorphisms in this gene has been reported to be associated with obesity and body weight in humans [34], and Fontanesi et al*.* [35] proposed *APOA2* as a candidate gene for back fat thickness in pigs. The genes *USP21* and *UFC1* are related to proteolysis gene ontology terms, and are members of Ubiquitin family gene. The ubiquitin system regulates virtually all aspects of cellular function [36], from regulation of gene expression to processes related to reticulum associated degradation of proteins, lysosomal degradation, protein degradation via the proteasome, cell signaling and DNA repair [37]. These processes are generally described as post-translational regulation of gene expression and may cause variation in phenotypes by influencing the levels of proteins and their activity in performing the biochemical processes [38]. Karisa et al. [38] identified a hub of *UBC* [ubiquitin C] associated with RFI in *Bos taurus*.

For FCR, four genomic regions explained >1% genetic variance (Table 3). The region on BTA18 at 15 Mb was also associated with FE (Additional file 1: Table S1). One functional candidate gene was selected within the genomic region on BTA15 at 58 Mb; lin7 homolog c (*LIN7C*). This gene has been assigned gene ontology terms associated with protein transport and localization (GO: 0008104). *LIN7C* gene plays has a hole on type-2 diabetes primarily through modulation of adiposity, and Maggie et al. [39] analyzed genetic variants in *LIN7C* related with obesity and type-2 diabetes in humans. The glial cell line-derived neurotrophic factor (*GDNF*) is annotated in the 36 Mb genomic region on BTA20. The protein encoded by this gene has been described as secreted from adipose cells [40]. The adipocytes secrete hormones with an important role in fat deposition and obesity [41]. The gene ontology terms associated to it are muscle system process (GO: 0003012) and positive regulation of nitrogen compounding metabolic process (GO: 0051173).

Four genomic regions explained >1% of genotypic variance for FE (Table 3).

Four genomic regions explained >1% of genetic variance for RFI (Table 3). The region on BTA18 at 14 Mb is between two regions that each explained more than 0.5% of genetic variance for other feed efficiency traits (FCR and FE, Additional file 1: Table S1). Two positional candidate genes related to proteolysis gene ontology terms (GO: 0006508; GO: 0006461) were selected in this region; dipeptidase 1 (*DEPP1*) and tubulin beta 3 (*TUBB3*). According to Richardson et al. [42] the protein degradation and protein turnover contribute to variation on RFI, and genes involved in these processes are good candidates for improving feed efficiency [43]. The associated region on BTA11 at 93 Mb harbors the prostaglandin-endoperoxide synthase 1 (*PTGS1). PTGS1* gene is involved with fatty acid metabolic process (GO: 0006631) and lipid biosynthetic (GO: 0008610). *PTGS1* is a key enzyme in prostaglandin biosynthesis, which are produced in different life stages of adipocytes [44]. Adipocyte differentiation and adipogenesis are important in terms of obesity and related to diabetes in mammalians [45].

Six 1 Mb genomic regions explained >1% of genetic variance for ME (Table 3). In addition to being associated with maintenance efficiency, two adjacent regions on BTA1, at 155 and 156 Mb, were also associated for ADG (Additional file 1: Table S1). A single gene that is associated to mouse development [46] was selected in this region; S. cerevisae, homolog (*DPH3*). The translation elongation factor 2 in eukaryotes (eEF-2) contains a unique posttranslational modified histidine residue, termed diphthamide [46]. *DPH3* is a small protein required for diphthamide biosynthesis and plays a role in eukaryotic protein complexes that is involved in multiple biological processes [47]*.* This gene has been assigned gene ontology terms associated with regulation of protein secretion (GO: 0050708) and transport (GO: 0051223). Five genomic regions explained >1% of genetic variance for EG (Table 3). The windows on BTA13 at 19 Mb and BTA20 at 42 Mb overlapped with regions associated with ME. The SNP window on BTA28 at 29 Mb harbors one gene with function related to calcium metabolism; calcium/calmodulin-dependent protein kinase II gamma (*CAMK2G*). Studies have implicated Ca^2+^ as a regulator of a variety of cellular functions, including cell cycle progression and proliferation, fertilization and early development [48]. *CAMK2G* gene has been implicated in the regulation of other biological processes, such as osteogenic differentiation and maintenance of vascular tone [48]. The *CAMK2G* is related to with cell cycle (GO: 0022402) and protein amino acid phosphorylation (GO: 0006468) gene ontology terms. Six genomic regions explained >1% of genetic variance for PEG (Table 3). The regions on BTA13 at 19 Mb and BTA14 at 83 Mb overlapped with regions for ME. One functional candidate gene have been selected in the window on BTA1 at 18 Mb; coxsackie virus and adenovirus receptor (*CXADR*). *CXADR* has been assigned gene ontology terms related to muscle organ development (GO: 0007517), regulation of organ growth (GO: 0046620), muscle tissue development (GO: 0060537) and regulation of developmental growth (GO: 0048638). Variants in *CXADR* gene were associated with blood pressure and obesity in humans [49]. *CXADR* plays a role in the electrical conduction of the heart, and it has also been reported to be associated with viral myocarditis and subsequent dilated cardiomyopathy, which is associated with high blood pressure [50]. An interesting gene is annotated on BTA26 at 46 Mb; adam metallopeptidase domain 12 (*ADAM12*). According to Coles et al. [51] *ADAM12* is an interesting gene in beef cattle due to its involvement in regulating remodeling of extracellular matrix, modulation of cell morphological changes, satellite cell activation, and ectodomain shedding during signaling of muscle and fat development [52,53]*. ADAM12* gene is involved in the regulation of myogenesis and adipogenesis in beef cattle [51]. Kim et al. [54] identified the *ADAM12* gene also as a regulator for TGF-β1. Transforming growth factor-β1 (TGF-β1) induces the differentiation of human adipose tissue–derived mesenchymal stem cells into smooth muscle cells.

Four genomic regions explained >1% of genetic variance for RGR (Table 3). Two genes with function related to skeletal growth have been chosen on BTA9 at 75 Mb; microtubule-associated protein 7 *(MAP7*) and peroxisomal biogenesis factor 7 *(PEX7). MAP7* has been assigned biological function related to growth (GO: 0040007), organ growth (GO: 0035265) and cytoskeleton organization (GO: 0007010). Skeletal muscle growth is a complex process [55], since the rate and extent of skeletal muscle growth depends on three factors: muscle protein synthesis; muscle protein degradation; and the number and size of skeletal muscle cells. The metabolic and functional characteristics of skeletal muscles are a result of the biochemical characteristics of the myofibers and the connective tissue matrix [56]. The basic unit of skeletal muscle in all metazoans is the multinucleate myofiber, within which individual nuclei are regularly positioned. *MAP7* gene is an essential regulator of myonuclear positioning required for skeletal muscle function in mammalians [57]. *PEX7* has also been assigned gene ontology terms associated to skeletal system development (GO: 0001501) and bone development (GO: 0060348). This gene was related to skeleton development in mouse [58].

The QTL regions described in this study harbor genes with biological functions related to immune and inflammatory responses, ionic and calcium metabolism, proteolysis, lipid and fatty acid metabolism, glycolysis, ubiquitin system, generation of energy, growth and development. Barendse et al. [23] in a study that utilized *Bos taurus* and *Bos indicus* steers reported 141 genetic regions associated with RFI. The described genes had biological functions also related to growth and development, immune and inflammation, protein turnover, ion channels and DNA binding proteins, fatty acid metabolism, ubiquitin system, and others additional process like transcription and translation, apoptosis, subcellular organelles, signal transduction and leptin signaling. Rolf et al. [22] in a GWAS for feed efficiency in Angus cattle also reported genes related to metabolic processes for growth and efficiency of energy utilization, such as cell growth and death, metabolic disorders and signal transduction. In a study with Nelore steers, Bonilha et al. [17] reported other physiological processes with an important role in feed efficiency; digestibility, feeding pattern, heat production, physical activity, stress, composition of gain and ruminal metabolism. Santana et al. [10] in recent GWAS for RFI and DMI with Nelore cattle found genes related to ionic transport, ionic exchange processes and leptin signaling. These results indicate that several physiological mechanisms maybe behind feed intake control in beef cattle. Although no functional experiment was done to support the implication of these genes on the phenotypes, these are the best candidates based on the methodology used to indicate genes within QTL regions.

Some genomic regions overlapped between the traits analyzed in this study. Regions on BTA9 overlapped between seven different efficiency traits; DMI, ADG, FE, ME, RGR, EG and PEG (Table 3, Additional file 1: Table S1). One region on BTA9 at 22 Mb overlapped between FE and ADG; and the other region at 85 Mb overlapped between FE, GE and ME. This chromosome harbors QTLs described for body weight in Angus cattle [59] and residual feed intake in Angus and Charolais cattle [24].

The BTA 1, 13, 14, 18 and 24 also contained regions that overlapped between the traits analyzed (Table 3, Additional file 1: Table S1). Two regions on BTA1 overlapped between ADG, ME and GE 156 Mb; and between PEG, ME and EG 180 Mb. The BTA1 has previously QTL described for body weight and feed conversion ratio in *Bos taurus* [59].

A single region on BTA13 19 Mb overlapped between ME, GE and PEG. The BTA13 has previously QTL described for carcass weight and body weight [59] in Angus and Charolais cattle [8].

On BTA14, one region at 83 Mb overlapped between 5 traits: FE, FCR, ME, EG and PEG. The BTA14 harbor already described QTL for body weight in Brangus cattle [60] and subcutaneous fat in Canchim cattle [61]. Other studies found SNPs associated with DMI in the BTA14 in beef cattle [22,62] and pigs [63].

For BTA18, the 15 Mb region overlapped between FCR and FE. This chromosome harbor QTL described for carcass weight in *Bos indicus* x *Bos taurus* cattle [64] and fat thickness in Angus cattle [59]. On BTA24, the 32 Mb region overlapped between two traits, DMI and RFI. The BTA24 has previously QTL described fat thickness for *Bos taurus* cattle [59]. These overlapped regions on BTA 1, 9, 13, 14, 18 and 24 seems to be important QTL regions for feed efficiency traits in this study.

The genomic regions controlling dry matter intake and residual feed intake in this study are somehow different from those in previous work. Santana et al. [10] found the BTA 4, 8, 14 and 21 associated to DMI and RFI in Nelore cattle. In this study, regions on BTA 9, 13 and 24, and on BTA 8, 11, 18 and 21 were identified for DMI and RFI, respectively. Only BTA 8 and 21 had a RFI QTL in both studies. These differences can be partially explained by differences in the experimental design and genetic basis of populations. Some of the discrepancies observed could be the result of hormonal environment effects on genes or QTLs, since in Santana et al. [10] both young bulls and steers were used, whereas in the present work there were only steers. Regarding to genetic basis, unrelatedness among sires of the different studies could lead to the differences observed.

Feed efficiency is a polygenic trait [9] characterized by complex interactions between cellular constituents (DNA, RNA) and proteins, influenced by multiple biological process [38]. Our results indicate several QTLs and genes control feed efficiency traits in Nelore cattle. Some QTL regions (BTA 3, 10, 11, 15, 20, 28) and some functional candidate genes identified have not been reported in public databases for feed efficiency traits in *Bos taurus* cattle, which indicates that feed efficiency in this Nelore population maybe is controlled by some specific regions. Discrepancies of SNP allele frequencies or the extent of linkage disequilibrium (LD]) of markers between taurine and indicine cattle could result in different marker effects being detected in different breeds [65]. Also, the relatively modest size of the population employed in this study may influence the results, and more studies with more Nelore populations will be required to validate this results.

According to Barendse et al. [23] genes affecting a particular trait will change in different species, and breed differences in performance and efficiency are well established [8]. Despite some difference between genes and genomic regions found in this study for *Bos indicus,* the physiological and metabolic processes of immune and inflammatory responses, ionic and calcium metabolism, proteolysis, glycolysis, lipid and fatty acid metabolism, ubiquitin system, generation of energy, growth and development related to feed efficiency seems to be similar between the two subspecies [10,22,23,25].

The studies of genomic regions and candidate genes associated with production traits paves the way to understanding the possible biological processes related to these traits, and most of these physiological mechanisms are still unclear. GWAS of complex traits for Nelore cattle breed is now emerging and they are important since has a little research reporting regions and genes associated to feed efficiency traits in this breed.

## Conclusions

Besides some previously reported genomic regions associated to feed efficiency in *Bos taurus,* the present genome wide association study identified several novel genomic regions, which indicates that feed efficiency is controlled by some specific regions in this Nelore population. Genes within these regions could be candidate genes for feed efficiency traits in Nelore cattle.

Once the genes and/or genomic segments that control feed efficiency related traits have been identified it should be possible to determine the biological mechanisms and the genetic basis underlying these traits. Thus, further studies with other Nelore population to validate this results will be required in order to implement selection for this trait in Nelore breeding programs in Brazil.

Since feeding is the main component of cattle production costs and Nelore is the breed of greatest economic importance in Brazil, the ability to identify and select for feed efficient animals should have a considerable economic impact. Development of molecular criteria for improving feed efficiency in Nelore should also contribute to reductions in the environmental impact of beef production in the tropics.

## Methods

Animals were handled and managed according to Institutional Animal Care and Use Committee Guidelines (Brazilian Agricultural Research Corporation – EMBRAPA, Brazil).

### Animals and phenotypic data

A total of 593 Nelore steers with average of 382.5 kg, offspring’s of 34 sires were used in this study. Sires were chosen to represent the main genealogies based on the information of the principal summaries of Brazilian Associations and to represent the average price of semen in use by Brazilian beef cattle farmers. Half-sib families were produced by artificial insemination of commercial and purebred Nelore dams. The range of the number of offspring per sire was 2 to 20. Calves were born on three different ranches, where they were raised to around 21 months old, before allocation to individual or collective pens where individual feed intake data were measured in a feedlot located in São Carlos, SP, Brazil; or in Campo Grande, MS, Brazil.

Animals were fed ad libitum twice daily, with refusals of 5% discarded daily. Diets contained 40% dry matter (DM) in the form of corn silage (trial 1) or sorghum silage (trial 2); crude protein at 13.5% (trial 1), 15.4% (trial 2); energy densities of 2.8 (trial 1) or 2.6 Mcal metabolizable energy per kg DM (trial 2), 60% DM of concentrate, which contained ground corn, soybean meal, cotton seed (only trial 1), soybean grain (only trial 2), soybean hull, limestone, mineral mixture, urea and monensin (Rumensin®). The adaptation period was approximately 28 days and individual dry matter intake (DMI) was measured for at least 70 days with non-fasted body weight (BW) measured every 14 days.

Individual dry matter intake (DMI, kg/d) was obtained by the difference between offer and refusal and average daily gain (ADG, kg/d) was estimated by regression of body weight (BW) on days on feed using PROC REG (SAS, 2010). Feed conversion ratio (FCR, kg/kg) was computed as the ratio of DMI to ADG (kg/d), where the inverse of this ratio was represented by feed efficiency (FE, kg/kg). Residual feed intake (RFI, kg/d) was computed as the residuals from regression of DMI on mid-test BW^0.75^ and ADG [2] using mixed models, where contemporary group (CG) was defined as feedlot location, year, animal origin and pen type (individual or collective), which were considered fixed effects by MIXED procedure (SAS, 2010). The partial efficiency of growth (PEG, kg/kg) that represents the energetic efficiency for ADG above maintenance was computed as the ratio of ADG to the difference between average daily DMI and expected DMI for maintenance (DMIm**)**, where DMIm was computed using the NRC 1996 maintenance requirement equations and Zinn and Shen [66] equations to estimate net energy of the diet for maintenance. The efficiency of gain (EG, kg/Mcal) was obtained by dividing ADG by metabolizable energy intake (Mcal/d). To calculate the relative growth rate (RGR, %/d) the equation was: RGR = 100*(log BW_final_ – log BW_initial_)/days of experiment [5]. The total number of animals (N) used for GWAS was slightly different between traits because of different availability of data.

### DNA extraction and genotypic data

Genomic DNA was extracted from blood samples [65]. Genotyping was performed at the United States Department of Agriculture Laboratory of Functional Genomics (ARS/USDA) with the BovineHD BeadChip, 770 K (Illumina, San Diego, CA). Genome Studio Data Analysis software (Illumina) was used to visualize SNP data and to perform initial analyses. The genotypes were recorded in Illumina A/B allele format and transformed to a value of 0, 1, or 2, representing the number of B alleles present. Missing genotypes represented less than 0.2% of total observations and were replaced with the average number of B alleles for that locus. SNPs with call rate ≥ 95% and minor allele frequency (MAF) ≥ 5% were used in the analyses. SNPs in sex chromosomes and not mapped in the *Bos taurus* UMD 3.1 assembly were excluded. A total of 449,363 SNP were utilized in this study.

### Descriptive statistics

Descriptive statistics for ADG, DMI, FCR, FE, RFI, ME, EG, PEG and RGR were performed by PROC MEANS of SAS [67]. The PROC UNIVARIATE was used to test and visualize normality of the data, and PROC MIXED used to test significant effects. The fixed effects for contemporary group (CG) included origin, pen type, year and location of feedlot, and age as a covariate, were used in the genomic association model.

### Genome wide association study

Bayesian approaches were developed to avoid false positives and overestimation of QTL effects [60,68-70]. The Bayes C methodology [71-73] was used to estimate the genetic and residual variances for use as priors in Bayes B. The Bayes C priors assumed genetic and residual variance equal to 1 and π = 0.9997. The GWAS between genotypes and phenotypes (ADG, DMI, FCR, FE, RFI, ME, GE, PEG and RGR) were undertaken with Bayes B, which analyzed all SNP data simultaneously and assumed a different genetic variance for each SNP locus [18,19,73], based on the model equation:

$$ \boldsymbol{y}=\mathbf{X}\boldsymbol{b} + {\displaystyle \sum_{j=1}^K\boldsymbol{a}j\ \beta \kern0.1em j\kern0.1em \delta \kern0.1em j} + \boldsymbol{e} $$

where; **y** is a vector of phenotypic values, ***X*** is an incidence matrix for fixed effects, ***b*** is a vector of fixed effects representing contemporary groups, *K* is the number of SNP loci (449,363), ***a****j* is the column vector representing the SNP covariate at locus *j* coded as the number of B alleles, *βj* is the random substitution effect for locus *j*, which conditional on σ^2^_*β*_ was assumed normally distributed *N* (0, σ^2^_*β*_ ) when *δj* = 1 but *βj* = 0 when *δj* = 0, with *δj* being a random 0/1 variable indicating the absence (with probability π) or presence (with probability 1-π) of locus *j* in the model, and ***e*** is a vector of random residual effects assumed normally distributed *N* (0, σ^2^_*e*_). The variance σ^2^_*β*_ (or σ^2^_*e*_) was a priori assumed to follow a scaled inverse Chi-square with *vβ* = 4 (or *ve* = 10) degrees of freedom and scale parameter *S*^2^_*β*_ (or *S*^2^_*e*_). The scale parameter for markers was derived as a function of the assumed known genetic variance of the population, based on the average SNP allele frequency and number of SNP assumed to have nonzero effects, based on π = 0.9997. The parameter π was 0.9997 chosen to fit fewer markers than animals. The GWAS was conducted with GenSel software [74], which uses MCMC methods to calculate posterior mean estimates of marker effects and variances.

The chains included 41,000 iterations with first 1,000 samples used for burn-in [19]. Markers effects from every 40th post burn-in iteration were used to characterize genomic merit for each animal for every 1 Mb window. In the Bayesian variable selection multiple-regression models with π = 0.9997 about 100–150 SNP markers were fitted simultaneously in each MCMC iteration. Inference of associations in these multiple-regression models was based on 1-Mb genomic windows rather than on single markers [68,69]. Genomic windows were constructed based on the chromosome and base pair positions denoted in a marker map file based on UMD-3.1 [68].

The SNP effects from every 40th post burn in iteration were used to obtain samples from the posterior distribution of the proportion of variance accounted for by each window from 1,000 MCMC samples of genomic merit for each animal following Onteru et al. [69] and Peters et al. [70]. In this study 2,527 SNP windows were used across the 29 chromosomes. The proportion of genetic variance explained by each window in any particular iteration was obtained by dividing the variance of the breeding values for the window by the variance of breeding value for whole genome in that iteration. Window genetic variance was computed by multiplying the number of alleles that represent the SNP covariates for each consecutive SNP in a window by their respective posterior means for substitution effects. The SNP windows that explained >1% of genetic variance from Bayes B analysis were considered as QTL associated with traits [60]. SNP windows that explained 0.5 to 1% of genetic variance are present as additional information in the supplemental material. The UMD-3.1 bovine assembly in Animal QTL database (http://www.animalgenome.org/QTLdb) and the Bovine QTL database (https://genome.ucsc.edu/) were used to search for QTLs previously described in the literature. Positional candidate genes were selected in 1 Mb windows that explained >1% of genetic variance [60] based on physiological and metabolic function. DAVID [75] and GO [37] were used to ascribe the functional classification of genes. No pathway analysis was conducted. Genes with biological function related to the trait and with support on literature were defined as positional and functional candidate genes.

### Availability of supporting data

The data set supporting the results of this article is included within the article (and its additional files). Genotypic data is available upon request depending on a signed declaration of exclusive research purpose.

## Additional files

Additional file 1:
**QTL regions associated with feed efficiency traits in Nelore cattle.**
Additional file 2:
**Manhattan plots of QTL regions associated with dry matter intake in Nelore cattle.**
Additional file 3:
**Manhattan plots of QTL regions associated with average daily gain in Nelore cattle.**
Additional file 4:
**Manhattan plots of QTL regions associated with feed conversion ratio in Nelore cattle.**
Additional file 5:
**Manhattan plots of QTL regions associated with feed efficiency in Nelore cattle.**
Additional file 6:
**Manhattan plots of QTL regions associated with residual feed intake in Nelore cattle.**
Additional file 7:
**Manhattan plots of QTL regions associated with maintenance efficiency in Nelore cattle.**
Additional file 8:
**Manhattan plots of QTL regions associated with partial efficiency of grow in Nelore cattle.**
Additional file 9:
**Manhattan plots of QTL regions associated with efficiency of gain in Nelore cattle.**
Additional file 10:
**Manhattan plots of QTL regions associated with relative growth rate in Nelore cattle.**

## Abbreviations

ADG: Average daily gain
BW: Body weight
DM: Dry matter
DMI: Dry matter intake
DMIm: Dry matter intake for maintenance
EG: Efficiency of gain
FCR: Feed-conversion ratio
FE: Feed efficiency
GWAS: Genome wide association study
ME: Maintenance efficiency
PEG: Partial efficiency of growth
RGR: Relative growth rate
RFI: Residual feed intake
