# Supplementary material for: Identification of genomic regions associated with feed efficiency in Nelore cattle
Source: BMC Genet. 2014 Sep 26;15:100. doi: 10.1186/s12863-014-0100-0 (PMC4198703; doi:10.1186/s12863-014-0100-0)
Supplement: Additional file 1: — QTL regions associated with feed efficiency traits in Nelore cattle. [file 12863_2014_100_MOESM1_ESM.docx]

**Identification of genomic regions associated with feed efficiency in Nelore cattle**

**Additional file 1 - QTL regions associated with feed efficiency traits in Nelore cattle**

QTL regions that explain > 0.5% of genetic variance for average daily gain (kg/d), dry matter intake (kg/d), feed conversion ratio (kg/kg), feed efficiency (kg/kg), residual feed intake (kg/d), maintenance efficiency (kg/kg), efficiency of gain (kg/Mcal), partial efficiency of growth and relative growth rate (%/d) in Nelore cattle.

| **Additional File 1: QTL regions associated with feed efficiency traits in Nelore cattle.** | | | | | |
| --- | --- | --- | --- | --- | --- |
| **Phenotype** | **SNP Window**  **(Start and end SNP)** | **Number SNP in Window** | **% Variance explained by SNP window** | **Chr** | **Map Position (UMD 3.1 bovine assembly)** |
| ADG | [rs110787372](http://www.ncbi.nlm.nih.gov/projects/SNP/snp_ref.cgi?rs=110787372)- [rs134954387](http://www.ncbi.nlm.nih.gov/projects/SNP/snp_ref.cgi?rs=134954387) | 206 | 0.73 | 24 | 33001958…33982585 |
|  | [rs133801254](http://www.ncbi.nlm.nih.gov/projects/SNP/snp_ref.cgi?rs=133801254)- [rs134385432](http://www.ncbi.nlm.nih.gov/projects/SNP/snp_ref.cgi?rs=134385432) | 138 | 0.59 | 9 | 54001734…54973309 |
|  | [rs110312247](http://www.ncbi.nlm.nih.gov/projects/SNP/snp_ref.cgi?rs=110312247)- [rs135563562](http://www.ncbi.nlm.nih.gov/projects/SNP/snp_ref.cgi?rs=135563562) | 223 | 0.55 | 16 | 29001388…29987410 |
|  | [rs136695261](http://www.ncbi.nlm.nih.gov/projects/SNP/snp_ref.cgi?rs=136695261)- [rs134921622](http://www.ncbi.nlm.nih.gov/projects/SNP/snp_ref.cgi?rs=134921622) | 265 | 0.55 | 1 | 155006277…155998842 |
|  | [rs137497028](http://www.ncbi.nlm.nih.gov/projects/SNP/snp_ref.cgi?rs=137497028)- [rs110943619](http://www.ncbi.nlm.nih.gov/projects/SNP/snp_ref.cgi?rs=110943619) | 157 | 0.55 | 3 | 103003289…103998628 |
|  | [rs110681959](http://www.ncbi.nlm.nih.gov/projects/SNP/snp_ref.cgi?rs=110681959)- [rs134417726](http://www.ncbi.nlm.nih.gov/projects/SNP/snp_ref.cgi?rs=134417726) | 181 | 0.52 | 8 | 82000756…82997174 |
|  | [rs41803460](http://www.ncbi.nlm.nih.gov/projects/SNP/snp_ref.cgi?rs=41803460)- [rs110671046](http://www.ncbi.nlm.nih.gov/projects/SNP/snp_ref.cgi?rs=110671046) | 243 | 0.50 | 16 | 35003481…35998905 |
|  | [rs133082754](http://www.ncbi.nlm.nih.gov/projects/SNP/snp_ref.cgi?rs=133082754)- [rs42902510](http://www.ncbi.nlm.nih.gov/projects/SNP/snp_ref.cgi?rs=42902510) | 149 | 0.50 | 1 | 151000965…151999589 |
|  |  |  |  |  |  |
| DMI | [rs137694547](http://www.ncbi.nlm.nih.gov/projects/SNP/snp_ref.cgi?rs=137694547)- [rs43071594](http://www.ncbi.nlm.nih.gov/projects/SNP/snp_ref.cgi?rs=43071594) | 189 | 0.95 | 8 | 97002787…97997099 |
|  | [rs109193786](http://www.ncbi.nlm.nih.gov/projects/SNP/snp_ref.cgi?rs=109193786)- [rs109735608](http://www.ncbi.nlm.nih.gov/projects/SNP/snp_ref.cgi?rs=109735608) | 215 | 0.90 | 2 | 65003697…65984693 |
|  | [rs43447821](http://www.ncbi.nlm.nih.gov/projects/SNP/snp_ref.cgi?rs=43447821) - [rs110661345](https://exchange.iastate.edu/owa/redir.aspx?C=KJmKAgtZLUSWYTSS19fSCxOMoNMZctAIwVKaaM3fUMZRaw_DS8sUfkk_CedSjId3_O-z4uHVqGg.&URL=http%3a%2f%2fwww.ncbi.nlm.nih.gov%2fprojects%2fSNP%2fsnp_ref.cgi%3frs%3d110661345) | 254 | 0.86 | 6 | 3001917…3994395 |
|  | [rs135072686](http://www.ncbi.nlm.nih.gov/projects/SNP/snp_ref.cgi?rs=135072686)-[rs136404079](http://www.ncbi.nlm.nih.gov/projects/SNP/snp_ref.cgi?rs=136404079) | 284 | 0.65 | 21 | 61003991…61993075 |
|  | [rs42576050](http://www.ncbi.nlm.nih.gov/projects/SNP/snp_ref.cgi?rs=42576050)-[rs41980496](http://www.ncbi.nlm.nih.gov/projects/SNP/snp_ref.cgi?rs=41980496) | 203 | 0.60 | 21 | 44046471…44996964 |
|  | [rs133145159](http://www.ncbi.nlm.nih.gov/projects/SNP/snp_ref.cgi?rs=133145159)*-*[rs136867776](http://www.ncbi.nlm.nih.gov/projects/SNP/snp_ref.cgi?rs=136867776) | 159 | 0.50 | 19 | 12000176…12997525 |
|  |  |  |  |  |  |
| FCR | [rs136132267](http://www.ncbi.nlm.nih.gov/projects/SNP/snp_ref.cgi?rs=136132267)-[rs132707578](http://www.ncbi.nlm.nih.gov/projects/SNP/snp_ref.cgi?rs=132707578) | 187 | 0.95 | 1 | 106001306…106994610 |
|  | [rs135559142](http://www.ncbi.nlm.nih.gov/projects/SNP/snp_ref.cgi?rs=135559142)-[rs110828671](http://www.ncbi.nlm.nih.gov/projects/SNP/snp_ref.cgi?rs=110828671) | 132 | 0.83 | 10 | 18238…993197 |
|  | [rs43328164](http://www.ncbi.nlm.nih.gov/projects/SNP/snp_ref.cgi?rs=43328164)-[rs134696015](http://www.ncbi.nlm.nih.gov/projects/SNP/snp_ref.cgi?rs=134696015) | 295 | 0.80 | 3 | 6009105…6999643 |
|  | [rs135793109](http://www.ncbi.nlm.nih.gov/projects/SNP/snp_ref.cgi?rs=135793109)-[rs134017120](http://www.ncbi.nlm.nih.gov/projects/SNP/snp_ref.cgi?rs=134017120) | 244 | 0.65 | 14 | 83000515…83999590 |
|  | [rs43311488](http://www.ncbi.nlm.nih.gov/projects/SNP/snp_ref.cgi?rs=43311488)-[rs135786810](http://www.ncbi.nlm.nih.gov/projects/SNP/snp_ref.cgi?rs=135786810) | 295 | 0.61 | 16 | 60001696…60997190 |
|  | [rs136127571](http://www.ncbi.nlm.nih.gov/projects/SNP/snp_ref.cgi?rs=136127571)-[rs41872478](http://www.ncbi.nlm.nih.gov/projects/SNP/snp_ref.cgi?rs=41872478) | 173 | 0.55 | 12 | 50022587…50999539 |
|  | [rs42945830](http://www.ncbi.nlm.nih.gov/projects/SNP/snp_ref.cgi?rs=42945830)-[rs137456474](http://www.ncbi.nlm.nih.gov/projects/SNP/snp_ref.cgi?rs=137456474) | 243 | 0.54 | 25 | 32000075…32992384 |
|  | [rs136086133](http://www.ncbi.nlm.nih.gov/projects/SNP/snp_ref.cgi?rs=136086133)-[rs136846608](http://www.ncbi.nlm.nih.gov/projects/SNP/snp_ref.cgi?rs=136846608) | 107 | 0.52 | 18 | 13004282…13994812 |
|  |  |  |  |  |  |
| FE | [rs109801168](http://www.ncbi.nlm.nih.gov/projects/SNP/snp_ref.cgi?rs=109801168) -[rs121919078](http://www.ncbi.nlm.nih.gov/projects/SNP/snp_ref.cgi?rs=121919078) | 141 | 0.89 | 16 | 33002827…33992046 |
|  | [rs133364656](http://www.ncbi.nlm.nih.gov/projects/SNP/snp_ref.cgi?rs=133364656) -[rs134251358](http://www.ncbi.nlm.nih.gov/projects/SNP/snp_ref.cgi?rs=134251358) | 137 | 0.82 | 2 | 2020914…2997466 |
|  | [rs135793109](http://www.ncbi.nlm.nih.gov/projects/SNP/snp_ref.cgi?rs=135793109)*-*[rs134017120](http://www.ncbi.nlm.nih.gov/projects/SNP/snp_ref.cgi?rs=134017120) | 244 | 0.79 | 14 | 83000515…83999590 |
|  | [rs41642281](http://www.ncbi.nlm.nih.gov/projects/SNP/snp_ref.cgi?rs=41642281) - [rs136649912](http://www.ncbi.nlm.nih.gov/projects/SNP/snp_ref.cgi?rs=136649912) | 241 | 0.77 | 15 | 34004680…34998425 |
|  | [rs110438768](http://www.ncbi.nlm.nih.gov/projects/SNP/snp_ref.cgi?rs=110438768) -[rs132904937](http://www.ncbi.nlm.nih.gov/projects/SNP/snp_ref.cgi?rs=132904937) | 196 | 0.71 | 3 | 62004226…62995709 |
|  | [rs110886051](http://www.ncbi.nlm.nih.gov/projects/SNP/snp_ref.cgi?rs=110886051) -[rs136158385](http://www.ncbi.nlm.nih.gov/projects/SNP/snp_ref.cgi?rs=136158385) | 132 | 0.65 | 9 | 85000924…85992426 |
|  | [rs132642498](http://www.ncbi.nlm.nih.gov/projects/SNP/snp_ref.cgi?rs=132642498) - [rs42290512](http://www.ncbi.nlm.nih.gov/projects/SNP/snp_ref.cgi?rs=42290512) | 195 | 0.59 | 20 | 42002534…42999156 |
|  | [rs110312247](http://www.ncbi.nlm.nih.gov/projects/SNP/snp_ref.cgi?rs=110312247) -[rs135563562](http://www.ncbi.nlm.nih.gov/projects/SNP/snp_ref.cgi?rs=135563562) | 223 | 0.57 | 16 | 29001388…29987410 |
|  | [rs109105703](http://www.ncbi.nlm.nih.gov/projects/SNP/snp_ref.cgi?rs=109105703) -[rs136356118](http://www.ncbi.nlm.nih.gov/projects/SNP/snp_ref.cgi?rs=136356118) | 189 | 0.55 | 18 | 15002281…15989210 |
|  | [rs133801254](http://www.ncbi.nlm.nih.gov/projects/SNP/snp_ref.cgi?rs=133801254) -[rs134385432](http://www.ncbi.nlm.nih.gov/projects/SNP/snp_ref.cgi?rs=134385432) | 138 | 0.53 | 19 | 54001734…54973309 |
|  |  |  |  |  |  |
| RFI | [rs135744174](http://www.ncbi.nlm.nih.gov/projects/SNP/snp_ref.cgi?rs=135744174)-[rs109110129](http://www.ncbi.nlm.nih.gov/projects/SNP/snp_ref.cgi?rs=109110129) | 160 | 0.73 | 2 | 111003171…111998250 |
|  | [rs134109723](http://www.ncbi.nlm.nih.gov/projects/SNP/snp_ref.cgi?rs=134109723)-[rs137285753](http://www.ncbi.nlm.nih.gov/projects/SNP/snp_ref.cgi?rs=137285753) | 132 | 0.69 | 5 | 79005067…79995258 |
|  | [rs43497129](http://www.ncbi.nlm.nih.gov/projects/SNP/snp_ref.cgi?rs=43497129)-[rs136390112](http://www.ncbi.nlm.nih.gov/projects/SNP/snp_ref.cgi?rs=136390112) | 148 | 0.68 | 6 | 119004594…119454666 |
|  | [rs137741468](http://www.ncbi.nlm.nih.gov/projects/SNP/snp_ref.cgi?rs=137741468)-[rs109288157](http://www.ncbi.nlm.nih.gov/projects/SNP/snp_ref.cgi?rs=109288157) | 192 | 0.67 | 26 | 10009703…10995721 |
|  | [rs133031353](http://www.ncbi.nlm.nih.gov/projects/SNP/snp_ref.cgi?rs=133031353)-[rs42739324](http://www.ncbi.nlm.nih.gov/projects/SNP/snp_ref.cgi?rs=42739324) | 237 | 0.67 | 24 | 32007830…32998471 |
|  | [rs110088745](http://www.ncbi.nlm.nih.gov/projects/SNP/snp_ref.cgi?rs=110088745)*-*[rs43549651](http://www.ncbi.nlm.nih.gov/projects/SNP/snp_ref.cgi?rs=43549651) | 233 | 0.62 | 8 | 40010769…40994687 |
|  | [rs135695322](http://www.ncbi.nlm.nih.gov/projects/SNP/snp_ref.cgi?rs=135695322)-[rs136588271](http://www.ncbi.nlm.nih.gov/projects/SNP/snp_ref.cgi?rs=136588271) | 283 | 0.59 | 15 | 73014830…73997794 |
|  | [rs135460402](http://www.ncbi.nlm.nih.gov/projects/SNP/snp_ref.cgi?rs=135460402)-[rs109526284](http://www.ncbi.nlm.nih.gov/projects/SNP/snp_ref.cgi?rs=109526284) | 232 | 0.56 | 19 | 5011545…5989303 |
|  |  |  |  |  |  |
| ME | [rs135553767](http://www.ncbi.nlm.nih.gov/projects/SNP/snp_ref.cgi?rs=135553767)-[rs133216097](http://www.ncbi.nlm.nih.gov/projects/SNP/snp_ref.cgi?rs=133216097) | 265 | 0.98 | 20 | 58002823…58996938 |
|  | [rs133644625](http://www.ncbi.nlm.nih.gov/projects/SNP/snp_ref.cgi?rs=133644625)*-*[rs137494465](http://www.ncbi.nlm.nih.gov/projects/SNP/snp_ref.cgi?rs=137494465) | 181 | 0.78 | 12 | 44013402…44986882 |
|  | [rs42066681](http://www.ncbi.nlm.nih.gov/projects/SNP/snp_ref.cgi?rs=42066681)-[rs133291391](http://www.ncbi.nlm.nih.gov/projects/SNP/snp_ref.cgi?rs=133291391) | 240 | 0.61 | 5 | 4010093…4995711 |
|  | [rs43109937](http://www.ncbi.nlm.nih.gov/projects/SNP/snp_ref.cgi?rs=43109937)-[rs135732317](http://www.ncbi.nlm.nih.gov/projects/SNP/snp_ref.cgi?rs=135732317) | 202 | 0.59 | 1 | 18002981…18998146 |
|  | [rs109372510](http://www.ncbi.nlm.nih.gov/projects/SNP/snp_ref.cgi?rs=109372510)-[rs134352492](http://www.ncbi.nlm.nih.gov/projects/SNP/snp_ref.cgi?rs=134352492) | 222 | 0.56 | 7 | 77004653…77998425 |
|  | [rs134352492](http://www.ncbi.nlm.nih.gov/projects/SNP/snp_ref.cgi?rs=134352492)-[rs133053098](http://www.ncbi.nlm.nih.gov/projects/SNP/snp_ref.cgi?rs=133053098) | 172 | 0.53 | 2 | 124023198…124997614 |
|  |  |  |  |  |  |
| EG | [rs137164093](http://www.ncbi.nlm.nih.gov/projects/SNP/snp_ref.cgi?rs=137164093)-[rs137788588](http://www.ncbi.nlm.nih.gov/projects/SNP/snp_ref.cgi?rs=137788588) | 158 | 0.91 | 3 | 55053110…55996246 |
|  | [rs135793109](http://www.ncbi.nlm.nih.gov/projects/SNP/snp_ref.cgi?rs=135793109)-[rs134017120](http://www.ncbi.nlm.nih.gov/projects/SNP/snp_ref.cgi?rs=134017120) | 244 | 0.8 | 14 | 83000515…83999590 |
|  | [rs137822645](http://www.ncbi.nlm.nih.gov/projects/SNP/snp_ref.cgi?rs=137822645)-[rs137079462](http://www.ncbi.nlm.nih.gov/projects/SNP/snp_ref.cgi?rs=137079462) | 178 | 0.76 | 20 | 56004883…56999854 |
|  | [rs134217291](http://www.ncbi.nlm.nih.gov/projects/SNP/snp_ref.cgi?rs=134217291)-[rs43586239](http://www.ncbi.nlm.nih.gov/projects/SNP/snp_ref.cgi?rs=43586239) | 245 | 0.72 | 1 | 156005880…156999077 |
|  | [rs43109937](http://www.ncbi.nlm.nih.gov/projects/SNP/snp_ref.cgi?rs=43109937)*-*[rs135732317](http://www.ncbi.nlm.nih.gov/projects/SNP/snp_ref.cgi?rs=135732317) | 202 | 0.69 | 1 | 18002981…18998146 |
|  | [rs110532506](http://www.ncbi.nlm.nih.gov/projects/SNP/snp_ref.cgi?rs=110532506)-[rs133928131](http://www.ncbi.nlm.nih.gov/projects/SNP/snp_ref.cgi?rs=133928131) | 154 | 0.65 | 10 | 17011165…17997362 |
|  | [rs134963634](http://www.ncbi.nlm.nih.gov/projects/SNP/snp_ref.cgi?rs=134963634)-[rs42390829](http://www.ncbi.nlm.nih.gov/projects/SNP/snp_ref.cgi?rs=42390829) | 207 | 0.64 | 20 | 55010178…55999450 |
|  | [rs136695261](http://www.ncbi.nlm.nih.gov/projects/SNP/snp_ref.cgi?rs=136695261)*-*[rs134921622](http://www.ncbi.nlm.nih.gov/projects/SNP/snp_ref.cgi?rs=134921622) | 265 | 0.62 | 1 | 155006277…155998842 |
|  | [rs42442541](http://www.ncbi.nlm.nih.gov/projects/SNP/snp_ref.cgi?rs=42442541)-[rs42102604](http://www.ncbi.nlm.nih.gov/projects/SNP/snp_ref.cgi?rs=42102604) | 324 | 0.55 | 26 | 46000238…46999583 |
|  | [rs110886051](http://www.ncbi.nlm.nih.gov/projects/SNP/snp_ref.cgi?rs=110886051)-[rs136158385](http://www.ncbi.nlm.nih.gov/projects/SNP/snp_ref.cgi?rs=136158385) | 132 | 0.54 | 9 | 85000924…85992426 |
|  | [rs110048374](http://www.ncbi.nlm.nih.gov/projects/SNP/snp_ref.cgi?rs=110048374)-[rs133053098](http://www.ncbi.nlm.nih.gov/projects/SNP/snp_ref.cgi?rs=133053098) | 172 | 0.53 | 2 | 124023198…124997614 |
|  | [rs137541109](http://www.ncbi.nlm.nih.gov/projects/SNP/snp_ref.cgi?rs=137541109)-[rs110015555](http://www.ncbi.nlm.nih.gov/projects/SNP/snp_ref.cgi?rs=110015555) | 219 | 0.52 | 5 | 82023942…82994350 |
|  | [rs134914044](http://www.ncbi.nlm.nih.gov/projects/SNP/snp_ref.cgi?rs=134914044)-[rs42277860](http://www.ncbi.nlm.nih.gov/projects/SNP/snp_ref.cgi?rs=42277860) | 203 | 0.51 | 1 | 21002744…21985704 |
|  |  |  |  |  |  |
| PEG | [rs110532506](http://www.ncbi.nlm.nih.gov/projects/SNP/snp_ref.cgi?rs=110532506)-[rs133928131](http://www.ncbi.nlm.nih.gov/projects/SNP/snp_ref.cgi?rs=133928131) | 154 | 1.01 | 10 | 17011165…17997362 |
|  | [rs42290518](http://www.ncbi.nlm.nih.gov/projects/SNP/snp_ref.cgi?rs=42290518)- [rs137428126](http://www.ncbi.nlm.nih.gov/projects/SNP/snp_ref.cgi?rs=137428126) | 158 | 0.85 | 20 | 43000253…43994737 |
|  | [rs41642281](http://www.ncbi.nlm.nih.gov/projects/SNP/snp_ref.cgi?rs=41642281)-[rs136649912](http://www.ncbi.nlm.nih.gov/projects/SNP/snp_ref.cgi?rs=136649912) | 241 | 0.78 | 15 | 34004680…34998425 |
|  | [rs134918768](http://www.ncbi.nlm.nih.gov/projects/SNP/snp_ref.cgi?rs=134918768)-[rs42901236](http://www.ncbi.nlm.nih.gov/projects/SNP/snp_ref.cgi?rs=42901236) | 154 | 0.69 | 20 | 44004196…44991729 |
|  | [rs132642498](http://www.ncbi.nlm.nih.gov/projects/SNP/snp_ref.cgi?rs=132642498)-[rs42290512](http://www.ncbi.nlm.nih.gov/projects/SNP/snp_ref.cgi?rs=42290512) | 195 | 0.69 | 20 | 42002534…42999156 |
|  | [rs110438768](http://www.ncbi.nlm.nih.gov/projects/SNP/snp_ref.cgi?rs=110438768)-[rs132904937](http://www.ncbi.nlm.nih.gov/projects/SNP/snp_ref.cgi?rs=132904937) | 196 | 0.67 | 3 | 62004226…62995709 |
|  | [rs43590097](http://www.ncbi.nlm.nih.gov/projects/SNP/snp_ref.cgi?rs=43590097)-[rs43593862](http://www.ncbi.nlm.nih.gov/projects/SNP/snp_ref.cgi?rs=43593862) | 208 | 0.63 | 9 | 34003997…34999679 |
|  | [rs110686871](http://www.ncbi.nlm.nih.gov/projects/SNP/snp_ref.cgi?rs=110686871)-[rs109738686](http://www.ncbi.nlm.nih.gov/projects/SNP/snp_ref.cgi?rs=109738686) | 126 | 0.58 | 10 | 18003127…18983183 |
|  | [rs135591942](http://www.ncbi.nlm.nih.gov/projects/SNP/snp_ref.cgi?rs=135591942)-[rs136061761](http://www.ncbi.nlm.nih.gov/projects/SNP/snp_ref.cgi?rs=136061761) | 139 | 0.56 | 20 | 46003628…46999429 |
|  | [rs43041698](http://www.ncbi.nlm.nih.gov/projects/SNP/snp_ref.cgi?rs=43041698)-[rs109491336](http://www.ncbi.nlm.nih.gov/projects/SNP/snp_ref.cgi?rs=109491336) | 275 | 0.54 | 20 | 40001507…40996382 |
|  |  |  |  |  |  |
| RGR | [rs42008483](http://www.ncbi.nlm.nih.gov/projects/SNP/snp_ref.cgi?rs=42008483)- [rs133648313](http://www.ncbi.nlm.nih.gov/projects/SNP/snp_ref.cgi?rs=133648313) | 127 | 0.83 | 22 | 46005889… 46999402 |
|  | [rs110944108](http://www.ncbi.nlm.nih.gov/projects/SNP/snp_ref.cgi?rs=110944108)- [rs133368945](http://www.ncbi.nlm.nih.gov/projects/SNP/snp_ref.cgi?rs=133368945) | 170 | 0.69 | 3 | 17007477… 17998304 |
|  | [rs109785180](http://www.ncbi.nlm.nih.gov/projects/SNP/snp_ref.cgi?rs=109785180)- [rs42314597](http://www.ncbi.nlm.nih.gov/projects/SNP/snp_ref.cgi?rs=42314597) | 181 | 0.66 | 7 | 28006905… 28999205 |
|  | [rs110244417](http://www.ncbi.nlm.nih.gov/projects/SNP/snp_ref.cgi?rs=110244417)- [rs42198814](http://www.ncbi.nlm.nih.gov/projects/SNP/snp_ref.cgi?rs=42198814) | 139 | 0.5 | 25 | 30000273… 30993420 |
| ADG: average daily gain (kg/d), DMI: dry matter intake (kg/d), FCR: feed conversion ratio (kg/kg), FE: feed efficiency (kg/kg), RFI: residual feed intake (kg/d), ME: maintenance efficiency (kg/kg), EG: efficiency of gain (kg/Mcal), PEG: partial efficiency of growth, RGR: relative growth rate (%/d). | | | | | |
