# Supplementary material for: Identification of genomic regions associated with feed efficiency in Nelore cattle
Source: BMC Genet. 2014 Sep 26;15:100. doi: 10.1186/s12863-014-0100-0 (PMC4198703; doi:10.1186/s12863-014-0100-0)
Supplement: Additional file 8: — Manhattan plots of QTL regions associated with partial efficiency of grow in Nelore cattle. [file 12863_2014_100_MOESM8_ESM.docx]

**Identification of genomic regions associated with feed efficiency in Nelore cattle**

**Additional file 8 - Manhattan plots of QTL regions associated with partial efficiency of grow in Nelore cattle**

Figure of Manhattan plots of QTL regions associated with partial efficiency of grow in Nelore cattle. The X-axis represents the chromosomes, and the Y-axis shows the proportion of genetic variance explained by SNP window from Bayes B analysis.


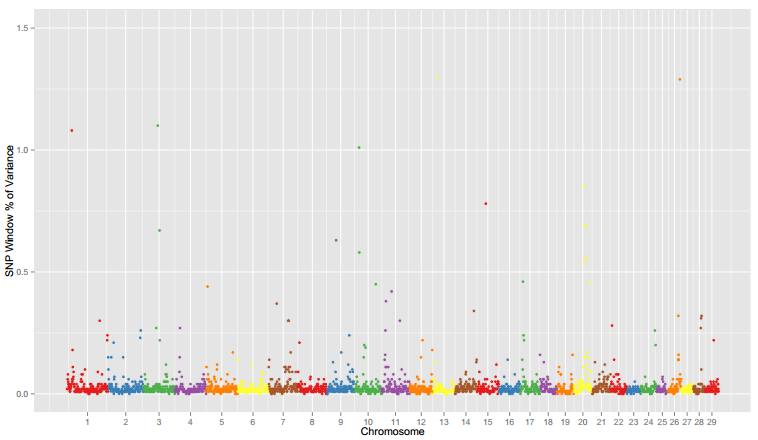


**Additional File 8.** Manhattan plots of QTL regions associated with partial efficiency of grow in Nelore cattle. The X-axis represents the chromosomes, and the Y-axis shows the proportion of genetic variance explained by SNP window from Bayes B analysis.
